# A genome-wide analysis of desferrioxamine mediated iron uptake in *Erwinia* spp. reveals genes exclusive of the Rosaceae infecting strains

**DOI:** 10.1038/s41598-019-39787-x

**Published:** 2019-02-26

**Authors:** Ivan Polsinelli, Luigimaria Borruso, Rosanna Caliandro, Luca Triboli, Alfonso Esposito, Stefano Benini

**Affiliations:** 1 Bioorganic Chemistry and Bio-Crystallography laboratory (B2Cl), Faculty of Science and Technology, Free University of Bolzano, Piazza Università 5, 39100 Bolzano, Italy; 2 Centre for Integrative Biology, University of Trento, via Sommarive n. 9, 38123 Povo, Trento Italy

## Abstract

*Erwinia amylovora* is the etiological agent of fire blight, a devastating disease which is a global threat to commercial apple and pear production. The *Erwinia* genus includes a wide range of different species belonging to plant pathogens, epiphytes and even opportunistic human pathogens. The aim of the present study is to understand, within the *Erwinia* genus, the genetic differences between phytopathogenic strains and those strains not reported to be phytopathogenic. The genes related to the hydroxamate siderophores iron uptake have been considered due to their potential druggability. In *E*. *amylovora* siderophore-mediated iron acquisition plays a relevant role in the progression of Fire blight. Here we analyzed the taxonomic relations within *Erwinia* genus and the relevance of the genes related to the siderophore-mediated iron uptake pathway. The results of this study highlight the presence of a well-defined sub-group of Rosaceae infecting species taxonomically and genetically related with a high number of conserved core genes. The analysis of the complete ferrioxamine transport system has led to the identification of two genes exclusively present in the Rosaceae infecting strains.

## Introduction

Fire blight is one of the major threat to Rosaceae with a potentially disastrous economic impact on apple and pear production^1^. With favorable environmental conditions, an outbreak can cause the loss of entire annual harvests. The etiological agent of the disease is *Erwinia amylovora*, one of the top ten known plant pathogens^2^. The *Erwinia* genus comprises species that are plant pathogens, non-pathogen, epiphytes, and even opportunistic human pathogens^3–5^. *E*. *amylovora* can infect a wide range of hosts, comprehensive of apple, pear, hawthorn, cotoneaster, rubus, etc^6^. Earlier comparative genomic studies, focused on virulence genes, suggest that *E*. *amylovora* host specificity and pathogenicity is driven by the absence or presence of certain genes^7^.

In living organisms, iron is a fundamental element, present as a cofactor in proteins and enzymes (e.g., iron-sulfur clusters, heme groups, etc.). A broad spectrum of biological relevant reactions are catalyzed by the reversible Fe(II)/Fe(III) redox pair^8^. The restriction on the availability of iron led to the development of highly selective systems for its acquisition, either directly (e.g. through transferrin, heme or hemoproteins) or indirectly, through hemophores or siderophores. In particular, siderophores are small molecules (usually < 1 kDa) that complex ferric iron and are among the strongest Fe(III) chelators^9^. Iron uptake is one of the main molecular pathways involved in the fire blight disease progression^10,11^. Not all *Erwinia* species are able to synthetize their own siderophores, suggesting that iron uptake is a potential niche adaptation factor^12^. *E*. *amylovora* mutants, with defective siderophore biosynthesis and uptake, showed an important reduction in their growth on apple flowers compared to the wild-type strain^13^. In *E*. *amylovora*, siderophore mediated iron uptake is dependent on desferrioxamines (DFOs)^14^: molecules consisting of alternating diamine and dicarboxylic acid building blocks linked by amide bonds. The major product of DFOs biosynthesis in *E*. *amylovora* is nocardamine (desferrioxamine E, DFO-E). Three proteins, namely DfoJ, DfoA and DfoC, are responsible for the biosynthesis of DFO-E starting from lysine^15,16^, these proteins are encoded by a single gene cluster, the *dfoJAC* operon. Together with the DFOs biosynthetic pathway proteins, the bacterium expresses a specific membrane receptor, called FoxR, necessary for the transport of ferrioxamine complexes across the outer membrane^17,18^. Transport across the bacterium inner membrane, possibly depends on the periplasmic binding protein-dependent ABC transporter complex FhuABCD, as reported for other hydroxamate siderophores of enteric bacteria e.g. in *Escherichia coli*^19–21^ and *Salmonella enterica*^22^. Ferrisiderophore complexes are very stable, and the mechanism of iron release can occur with three different mechanisms: hydrolysis of the siderophore, proton-assisted dissociation of the complex, and reduction of the metal center^23^. The lack of specific hydrolases in *E*. *amylovora* and the incompatibility of the cytoplasmic pH with the proton-assisted dissociation suggests that the iron is released after its reduction (ferrioxiamine has a lower affinity for Fe(II) than for Fe(III)), as in *E*. *coli*^24^.

The aim of the present study is to understand the genetic differences within *Erwinia* spp. between the species reported to be phytopathogenic and the one never reported to be correlated to plant diseases (here considered as non-phytopathogenic). A special focus is dedicated at the genes related to the hydroxamate sidephores iron uptake. We used a comparative genomic approach and selected a specific dataset of *Erwinia* spp. covering 11 genomes from phytophatogenic species, and 8 genomes from non-pathogenic. The genome selection has been limited to the available ones completed and correctly annotated. Average Nucleotide Identity (ANI), phylogenetic inference based on conserved marker genes, pangenomic analysis and molecular diversity analysis have been performed to get insights about the relevance of siderophore mediated iron acquisition in the evolution of pathogens’ hosts selectivity and virulence.

## Results

In order to work on a balanced dataset of the *Erwinia* genus, 11 plant pathogens and 8 non-pathogens genomes were selected among the sequenced genomes in *Erwinia* (Table 1). The genome selection has been limited to the ones suitable for the analysis (see Methods section). Seven of the 19 genomes examined have been reported to be Rosaceae infecting pathogens, belonging to 4 different species: *E*. *amylovora*, *E*. *pyrifoliae*, *Erwinia sp* Ejp617 and *E*. *piriflorinigrans*.

**Table 1:** List of the genomes used for this study.

Strain	Accession number	Habitat/host	Plant pathogenicity
*E*. *amylovora* CFBP1430^*,•^	GCA_000091565.1	*Crataegus* (hawthorn)	Pathogen of Rosaceae^26^
*E*. *amylovora* ATCC49946^•^	GCA_000027205.1	*Malus* sp. (apple tree)	Pathogen of Rosaceae^26^
*E*. *amylovora* E-2^•^	GCA_002803865.1	*Malus* sp. (apple tree)	Pathogen of Rosaceae^26,27^
*E*. *pyrifoliae* Ep1/96^•^	GCA_000027265.1	*Pyrus pyrofolia* (asian pear tree/nashi)	Pathogen of *Pyrus pyrifolia*^30^
*E*. *pyrifoliae* DSM-12163^•^	GCA_000026985.1	*Pyrus pyrifolia* (asian pear tree/nashi)	Pathogen of *Pyrus pyrifolia*^28^
*Erwinia sp*. Ejp617^•^	GCA_000165815.1	*Pyrus pyrifolia* (asian pear tree/nashi)	Pathogen of *Pyrus pyrifolia*^29^
*E*. *piriflorinigrans* CFBP-5888^•^	GCA_001050515.1	*Pyrus communis* (pear tree)	Pathogen of *Pyrus communis*^25^
*E*. *tasmaniensis* ET1/99	GCA_000026185.1	*Malus* sp. (apple tree)	Non-pathogen^30,47^
*E*. *billingiae* OSU19-1	GCF_001269445.1	*Pyrus communis* (pear tree)	Non-pathogen^48^
*E*. *billingiae* Eb661	GCA_000196615.1	*Malus* sp. (apple tree)	Non-pathogen^47^
*E*. *toletana* DAPP-PG-7351	GCA_000336255.1	*Olea* sp. (olive tree)	^a^Associated to the pathogen of *Olea* sp.^49^
*E*. *Olea*e DAPP-PG531	GCA_000770305.1	*Olea europaea* (olive tree)	Non-pathogen^50,51^
*E*. *tracheiphila* BuffGH	GCA_000975275.1	*Cucurbita pepo* ssp. Texana (squash plant)	Pathogen of Cucurbitaceae^52^
*E*. *tracheiphila* PSU-1	GCA_000404125.1	*Cucurbita pepo* ssp. Texana (squash plant)	Pathogen of Cucurbitaceae^52^
*E*. *mallotivora* BT-MARDI	GCA_000590885.1	*Carica* sp. (papaya tree)	Pathogen of *Carica* sp.^53^
*E*. *persicina* NBRC-102418	GCA_001571305.1	*Piezodorus guildinii* (guts of redbanded stink bug) and Leguminosae (legume plants)	Pathogen of Leguminosae^54,55^
*E*. *iniecta* B149	GCA_001267545.1	*Diuraphis noxia* (wheat aphid)	Non-pathogen^56^
*E*. *iniecta* B120	GCA_001267535.1	*Diuraphis noxia* (wheat aphid)	Non-pathogen^56^
*E*. *gerundensis* EM595	GCA_001517405.1	*Pyrus communis* (pear tree)	Non-pathogen^57^

^•^These strains are Rosaceae infecting pathogen.

^*^E. amylovora CFBP1430 is the reference genome where all the DNA gene sequences were extracted.

^a^Found on olive knots caused by *Pseudomonas savastanoi* pv. savastanoi. The presence of *E. toletana* is correlated with the virulence of the disease suggesting a possible interactions with *P. savastanoi* pv. savastanoi.

### Average Nucleotides Identity and Phylogenetic analysis

**Figure 1:**
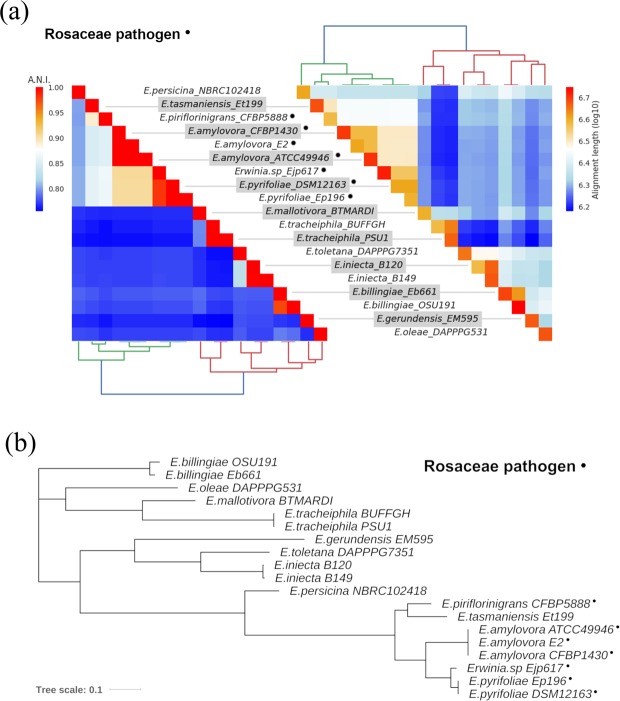
Relations within the selected *Erwinia* species. (**a**) Heatmap showing ANI analysis of the genomes: on the left the ANI values, on the right the length of the alignment. (**b**) PhyloPhlAn analysis: the phylogenetic tree was built on a dataset consisting of the concatenation of 400 universally conserved proteins. The phylogenetic distance is represented by the branch length.

The ANI values and the phylogenetic analysis consistently highlighted a clear division between the Rosaceae infecting pathogens (RIP) and the other species (Non Rosaceae infecting pathogens, NRIP). The genomes of the RIP group show higher similarity, with pairwise ANI values always higher than the ones from the other species and a large portion of genome aligned (above 6Mbp). Other species instead, had lower pairwise ANI values, also when the alignment lengths was comparable to the ones in the pathogenic species (Fig. 1a). Furthermore, the PhyloPhAn tree (Fig. 1b) reports a shorter phylogenetic distance between RIP species compared to the NRIP species. The tree topology is slightly different from the one in Fig. 1a, however the large basal split between RIP and NRIP is conserved. *Erwinia tasmaniensis* is an exception: in fact, although it has never been reported to be a pathogen, it shows a high genome similarity with the RIP group.

### Core and accessory genome in *Erwinia*

**Figure 2:**
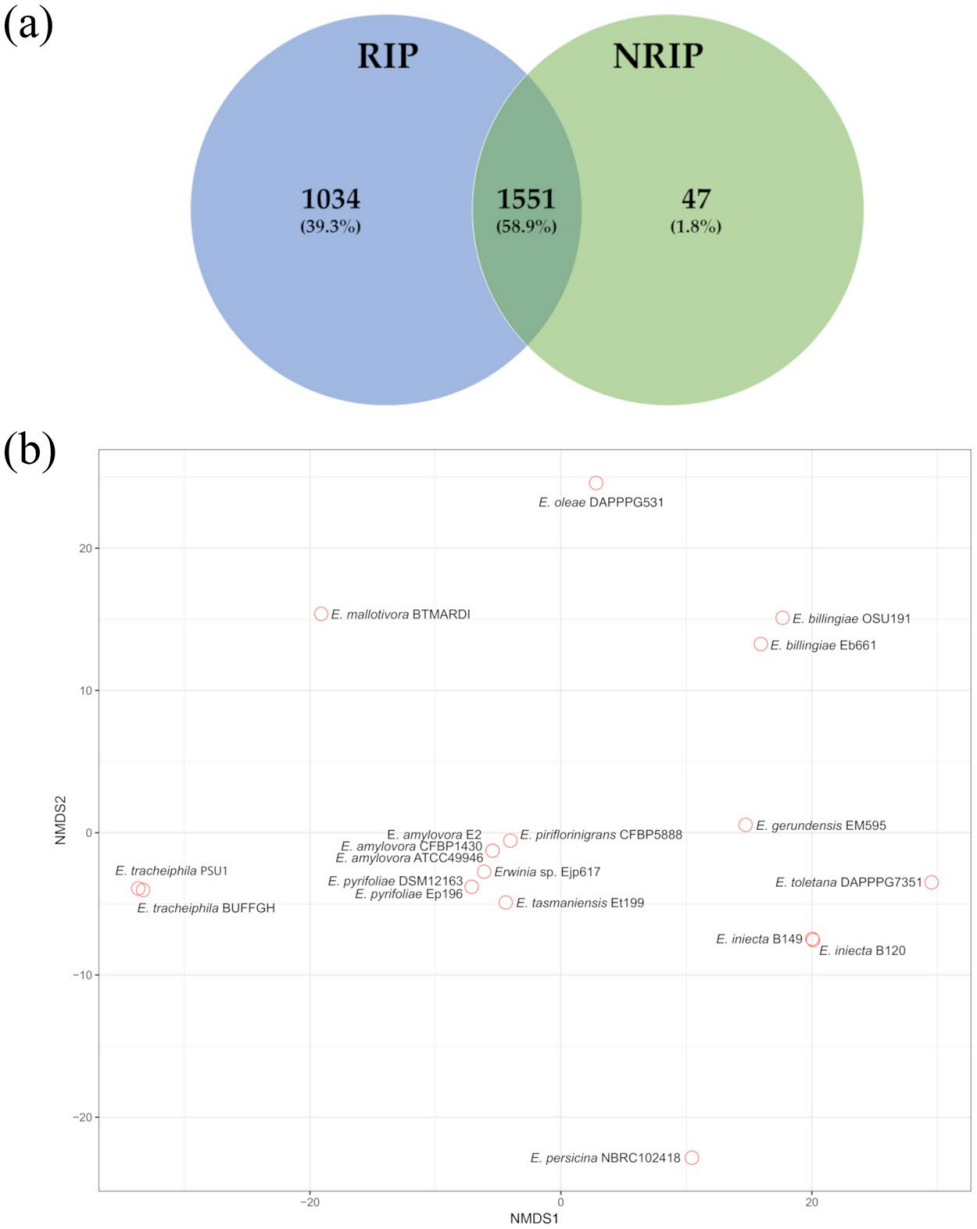
Pangenome analysis. (**a**) Venn diagram of the core genes shared within the two groups in *Erwinia* (RIP vs NRIP) (**b**) Non-metric MultiDimensional Scaling (NMDS): the plot is computed from the presence/absence matrix of all protein clusters in the genomes.

Clusters of orthologous proteins were created using Anvi’o, a binary matrix including the presence of genes in the different genomes was exported to perfom a pangenomic study. The core genome shared between RIP and NRIP is plotted in the Fig. 2a. A total of 1551 genes (58.9% of the core genes) are shared between the two groups (RIP and NRIP, hard-core genes), the number of soft-core genes (i.e. core within one of the two groups, but not in the other), is 1034 (39.3%) and 47 (1.8%) in RIP and NRIP, respectively. The multivariate analysis plot (Fig. 2b) displays patterns that are coherent with the findings of our phylogenetic analysis. All the RIP and *E*. *tasmaniensis* are concentrated in a closer space forming a narrow cluster in the center of the plot. The other species are scattered in both directions along the NMDS1 and NMDS2 axes within the plot without forming any distinguishable cluster.

### Presence/absence of genes related to hydroxamate siderophores iron uptake and polymorphism

**Figure 3:**
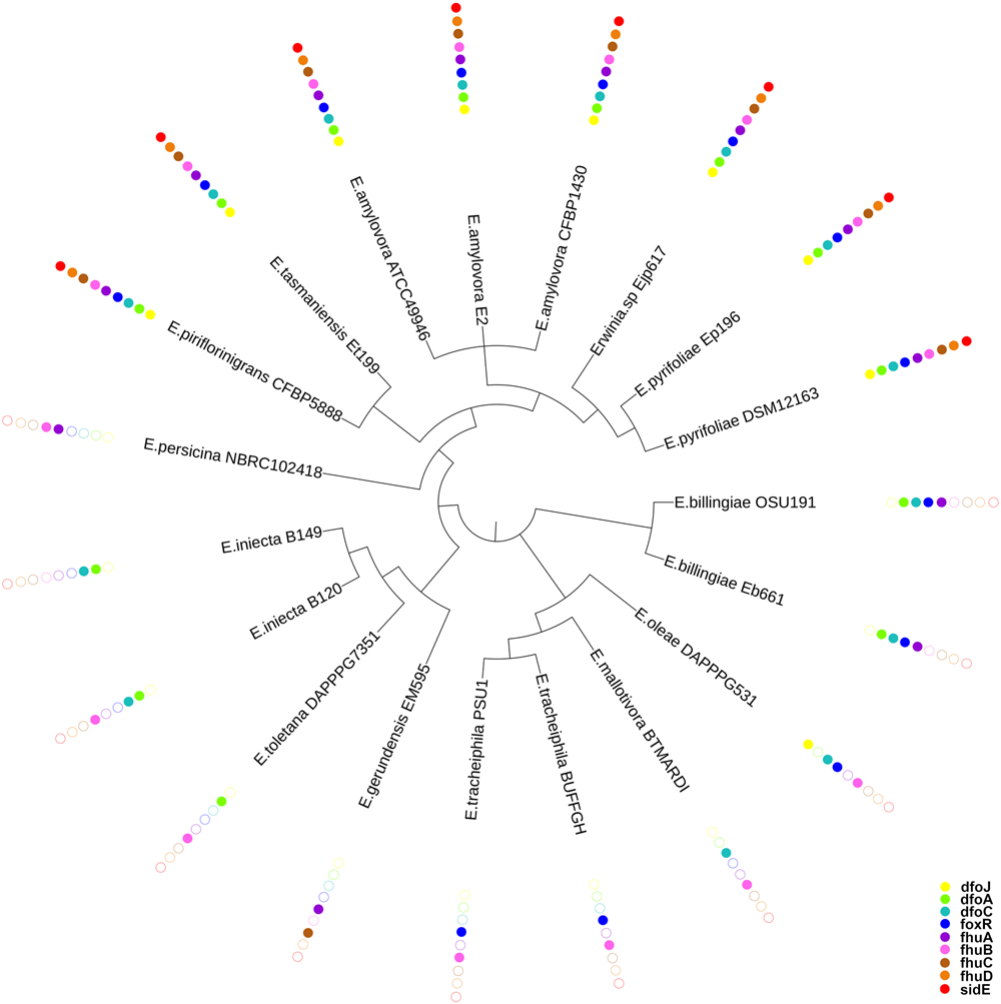
Representation of the genes presence according to the tree generated from PhyloPhlAn analysis (branch length are not shown in this figure to better visualize the topology of the tree). The dots represent the presence of a complete CDS for each gene.

Table 2 summarizes the presence/absence of coding sequences (CDS) for genes related to hydroxamate siderophores iron uptake in the 19 *Erwinia* genomes taken into account. As shown in Table 2, 9 genes have been considered as marker. All the marker CDS have been found in the RIP and in *E*. *tasmaniensis*; in NRIP species the number of the CDS (found with a complete coding sequence) of interest ranged from 2 to 4 (Table 2). The most prevalent gene is *fhuB* (coding part of the hydroxamate import system), found in 15 out of 19 strains, followed by *dfoC*, *dfoA* and *foxR* (synthesis and transport of ferrioxamine). The least present are *fhuD* (periplasmic binding protein) and *sidE* (siderophores utilization protein), both found only in RIP and *E*. *tasmaniensis*. The combination of the PhyloPhlAn analysis and the CDS presence is represented in Fig. 3. A monophyletic clade constituted by all the RIP and *E*. *tasmaniensis* contains all the CDS screened. Moreover, the CDS for *fhuD* (in orange) and *sidE* (in red) can be found only in this clade. The CDS for *fhuB* (in pink) is almost ubiquitous but it is absent in *E*. *gerundensis*, in one of the *E*. *iniecta* (*E*. *iniecta* B149) and in both *E*. *billingiae*.

**Table 2:** Distribution of the marker genes in the genomes analyzed. The presence of a CDS for the marker is represented as a black dot.

Organisms	*fhuB*	*dfoC*	*dfoA*	*foxR*	*fhuA*	*dfoJ*	*fhuC*	*fhuD*	*sidE*	Total markers
*E*. *amylovora* CFBP1430^•^	•	•	•	•	•	•	•	•	•	9
*E*. *amylovora* ATCC49946^•^	•	•	•	•	•	•	•	•	•	9
*E*. *amylovora* E-2^•^	•	•	•	•	•	•	•	•	•	9
*E*. *piriflorinigrans* CFBP-5888^•^	•	•	•	•	•	•	•	•	•	9
*E*. *pyrifoliae* DSM-12163^•^	•	•	•	•	•	•	•	•	•	9
*E*. *pyrifoliae* Ep1/96^•^	•	•	•	•	•	•	•	•	•	9
*Erwinia*.sp Ejp617^•^	•	•	•	•	•	•	•	•	•	9
*E*. *tasmaniensis* Et1/99	•	•	•	•	•	•	•	•	•	9
*E*. *billingiae* Eb661		•	•	•	•					4
*E*. *billingiae* OSU19-1		•	•	•	•					4
*E*. *oleae* DAPP-PG531	•	•		•		•				4
*E*. *iniecta* B120	•	•	•							3
*E*. *iniecta* B149		•	•							2
*E*. *gerundensis* EM595					•		•			2
*E*. *mallotivora* BT-MARDI	•	•								2
*E*. *persicina* NBRC-102418	•				•					2
*E*. *toletana* DAPP-PG-7351	•		•							2
*E*. *tracheiphila* BuffGH	•			•						2
*E*. *tracheiphila* PSU-1	•			•						2
**Total genomes**	15	14	13	13	12	9	9	8	8	

^•^These strains are Rosaceae infecting pathogen.

#### *dfoA*

**Figure 4:**
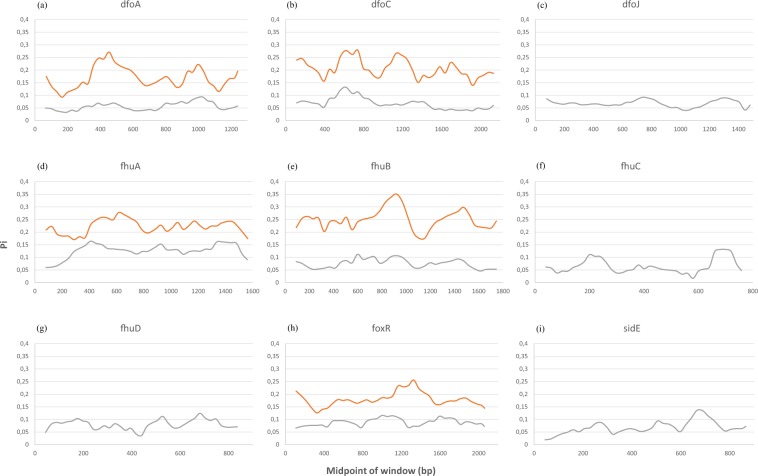
DNA polymorphism analysis of the genes involved in the siderophore mediated iron uptake within the two populations (RIP vs NRIP). RIP in grey, NRIP in orange.

A complete CDS for the gene *dfoA* was found in all RIP, in *E*. *tasmaniensis*, both strain of *E*. *iniecta*, *E*. *toletana* and one of the two *E*. *billingiae* strains (EB661). The other *E*. *billingiae* strain (OSU 191) missed an in-frame start codon. From none of the remaining species was possible to retrieve the homologous gene neither through blast, nor through the annotated genbank file. In RIP, the gene is conserved, with a Pi value below 0.1, whereas in NRIP there is a highly variable region around 400 bp. Despite the lower conserved of *dfoA* gene in NRIP, there are two noticeable conserved regions around 200 bp and 1100 bp (Fig. 4a).

#### *dfoC*

A complete CDS for *dfoC* was found in all the RIP, in *E*. *tasmaniensis*, in both *E*. *billingiae* and *E*. *iniecta*, in *E*. *olea* and *E*. *mallotivora*. It was not possible to retrieve the CDS from the annotated file. A remarkable feature of this alignment was a large (12 nt), in-frame insertion found in the two *E*. *iniecta* strains and *E*. *toletana*. In RIP, the gene is very conserved and in particular the region around 400 bp and the last 500 nucleotides. In the NRIP, the gene is generally less conserved than in RIP, with conserved 3 areas (regions around 400 bp, 1300 bp and 2000 bp, Fig. 4b). The variability pattern in this gene show a peak of variation at 1200 bp in NRIP that was not present in RIP.

#### *dfoJ*

A complete CDS was found using blast for all *E*. *amylovora*, the two *E*. *pyrifoliae*, *Erwinia sp*. Ejp617, *E*. *pyriflorinigrans* and *E*. *tasmaniensis*. It should be noted that from the genbank file, a feature annotated as *dfoJ* was present only in one out of three *E*. *amylovora* genomes (CFBP1430) and one *E*. *pyrifoliae* (DSM12163). A sequence with high identity with the *dfoJ* gene was also found in *E*. *oleae*, however, this gene was lacking an in-frame stop codon, even after manual inspection of the 5′ downstream region. The *dfoJ* gene was found only in RIP, and it display a relatively low value of Pi all along the sequence compared to the other genes (Fig. 4c).

#### *fhuA*

This gene was annotated only in two out of three *E*. *amylovora* strains (ATCC49946 and CFBP1430), and in the two *E*. *pyrifoliae*. However, a complete coding sequence was found in all *E*. *amylovora* and *E*. *pyrifoliae*, and in *Erwinia* sp., *E*. *tasmaniensis*, *E*. *persicina*, *E*. *piriflorinigrans*, both *E*. *billingiae* and *E*. *gerundensis*. The latter featured a TGA (opal) stop codon, whereas all other species have a TAA (ochre). As for the previous genes, the polymorphism is higher in NRIP than in RIP (Fig. 4d). This gene had a peak of variation in RIP in correspondence of a conserved region of NRIP, at 400 bp.

#### *fhuB*

Similar to *fhuA*, this gene was annotated in the same genomes, but found through blast in 16 out of 19 genomes (leaving out only the two *E*. *billingiae* and *E*. *gerundensis*). For *E*. *iniecta* B149, however, it was not possible to retrieve the whole species due to the fragmentation of the assembly (the 5′ end was cut by a stretch of “N”). Therefore, for the subsequent analysis, *fhuB* sequences for this genome was removed. The differences in the degree of polymorphism between the two populations (RIP vs NRIP) is evident (Fig. 4e), with maximum in the regions around 900 bp and 1500 bp.

#### *fhuC*

This was detected in all rosaceae infecting strains, in *E*. *tasmaniensis* and *E*. *gerundensis* (that once more had a TGA stop codon, and the most diverging sequence), although it was annotated only in the same four strains as *fhuA* and *fhuB*. The gene is conserved in RIP, with slightly higher polymorphism in the last 150 nucleotides (Fig. 4f).

#### *fhuD*

This was detected in all Rosaceae-infecting strains and in *E*. *tasmaniensis*, although it was annotated only in the same four strains as *fhuA* and *fhuB*. The gene is conserved in RIP (Fig. 4g).

#### *foxR*

This gene was detected in all RIP plus the two *E*. *billingiae*, the two *E*. *tracheiphila*, *E*. *oleae*, *E*. *tasmaniensis*. It was annotated in the genbank files of the two *E*. *pyrifoliae* and *E*. *amylovora* CFBP1430. The gene polymorphism is higher in NRIP than in RIP (Fig. 4h). In RIP, the most conserved regions are around 400 bp, 750 bp and 1300 bp, while in NRIP the most conserved regions are at 300 bp and 1600 bp. Once again, a peak of nucleotide variation in NRIP was detected in correspondence of a conserved region in RIP, at 1300 bp.

#### *sidE*

This gene was detected in all Rosaceae-infecting strains, plus *E*.*tasmaniensis*. In RIP, the gene is overall conserved, with a small peak at 700 bp (Fig. 4i).

## Discussion

The *Erwinia* genus includes phytopathogens affecting Rosaceae relevant in fruit production (e.g., *Malus* sp. and *Pyrus* sp.). In *E*. *amylovora*, the ferrioxamine biosynthesis and transport has been reported to be involved in the progress of the Fire blight disease^10–12,15,16^ which makes it a worth studying target pathway. In this work, a specific dataset of *Erwinia* spp. has been analyzed comparing at first the whole genome, then a group of conserved proteins, and at last focusing on specific siderophore genes. The ANI (Fig. 1a), the phylogenetic analysis (Fig. 1b), the pangenome and multivariate analysis based on the presence/absence of all genes (Fig. 2b) are coherent. The whole analysis highlights that a defined group of species is present in the heterogeneous *Erwinia* genus. All the species in this group, with the exception of *E*. *tasmaniensis*^25^, have been reported to be Rosaceae infecting pathogens^25–29^. Our results support that the lack of only few specific genes (involved in the biosynthesis and regulation of harpins, effectors and amylovoran) hints for a possible non-pathogenicity of *E*. *tasmaniensis* ET1/99 as suggested by Borruso *et al*.^7^ and others previously^30–32^. *Erwinia* spp. Ejp617 in all analysis clusters very close to the group of *E*. *pyrifoliae* although not as ingroup. According to our results *Erwinia* spp. Ejp617 isolated in Japan^28^ could be assigned within the species *E*. *pyrifoliae* as has been done in other reports^28,33^. All phylogenetic analysis (Figs 1 and 2b) suggest a different evolutionary rate within the RIP. These difference can be appreciated by looking at the branch length in Fig. 1b: the distance between *E*. *amylovora*, *E*. *pyrifoliae*, *Erwinia sp* Ejp617, *E*. *piriflorinigrans* and *E*. *tasmaniensis* is shorter than any distances among other species. The number of soft-core genes in the two groups is very different (Fig. 2a), suggesting a high level of host-adaptation required in the RIP for infecting the plant. The genomic similarity of RIP is revealed by the selected marker genes. The high level of host-adaptation is reflected in the iron uptake system, where the Pi value/polymorphism for all the genes from NRIP species is higher than in RIP (Fig. 4). Moreover, there are some contrasting nucleotide positions (e.g. 1200 bp in *dfoC* and 400 bp in *fhuA*) where there is a maximum of Pi value for one population and minimum for the other one. These regions could play a key role in the enzyme function and/or be part of the active site. Complete CDS for ferroxiamine siderophore biosynthesis (*dfoJAC*), outer membrane receptors (*fhuA* and *foxR*), periplasmic binding protein (*fhuD*), ABC cassette type receptor components (*fhuB* and *fhuC*) and siderophore utilization protein (*sidE*) are always present in all the Rosaceae infecting *Erwinia* (plus *E*. *tasmaniensis*). The role of ferrioxamine synthesis and *foxR* receptor in the disease has been previously reported^13,14,17,18^ and accordingly to our results, the complete transport system is relevant as well. Moreover, while some genes of the biosynthesis and the receptors are shared between RIP and NRIP, the genes coding for the periplasmic binding protein (*fhuD*) and the siderophore utilization protein (*sidE*) are strictly conserved in the RIP (Fig. 3). According to the PhyloPhlAn analysis, the epiphytic bacteria *E*. *billingiae* is the farthest *Erwinia* in respect of the RIP. However, it shares both the outer membrane receptors for hydroxamate siderophore and 2 out of 3 enzyme required for the ferrioxamine biosynthesis. *E*. *billingiae* tends to invade plants necrotic tissue^34,35^. *E*. *billingiae* could withdraw the intermediates and the ferrioxamine from the RIP, slowing down its growth, while scavenging the iron available in the plant tissues necrotized by pathogens. This behavior suggests a possible symbiosis/antagonism with the RIP^36,37^ even in the synthesis of siderophores. The absence of the almost ubiquitous gene *fhuB* from *E*. *billingiae*, *E*. *gerundensis*, and *E*. *iniecta* B149 could be explained with two hypotheses: i) it is possible that the gene, present in the ancestor, has been lost by the two derived species; or ii) a fragmented assembly may result in a missing gene. The latter is the most probable for *E*. *iniecta* B149, because the assembly consists of 121 contigs, whereas *E*. *gerudensis* consists of 3 contigs. However, in both *E*. *billingiae* and in *E*. *gerundensis*, the gene was most likely lost.

## Methods

In order to have a balanced dataset of the *Erwinia* genus (11 plant pathogens *vs* 8 non-pathogens) genomes to be screened were selected among the sequenced genomes in *Erwinia* according to the list in Borruso *et al*.^7^, both fasta and genbank format were downloaded from genbank on the 14th of February 2018. To avoid biases due to the much larger number of sequenced *E*. *amylovora* genomes we selected the three genomes with the highest completion score, namely *E*. *amylovora* strain CFBP1430, strain 49946 and strain E-2. Eventually, 19 genomes, spanning 13 species, were selected. Seven genomes (in four species), belonged to species known to infect Rosaceae, the remaining 12, spanned 9 species in which this phenotype is not reported (Table 1). Siderophore mediated iron uptake genes of *Erwinia* spp. were selected for their relevance: *dfoA* (ordered locus name EAMY_3239), *dfoC* (EAMY_3240), *dfoJ* (EAMY_3238), *fhuA* (annotated as 3 fragments: EAMY_2775, EAMY_2776 and EAMY_2777), *fhuB* (EAMY_2772), *fhuC* (EAMY_2774), *fhuD* (EAMY_2773), *foxR* (EAMY_3241), *sidE* (EAMY_3562). The gene sequences from *E*. *amylovora* CFBP1430 have been used as reference. A first genome-wide comparison was done using the Average Nucleotide Identity (ANI), using the software PYANI^38^, and the output was elaborated using the software DiMHepy (https://github.com/lucaTriboli/DiMHepy). A phylogenetic tree was built on the concatenation of 400 conserved proteins using PhyloPhlAn^39^, and the tree was drawn and annotated (e.g. presence absence of genes was reported on each leaf of the tree) using IToL^40^. To infer the number of shared protein clusters, genomes were imported in Anvi’o^41^, the pipeline followed the standard pangenomic workflow with all default parameters. A binary matrix containing all protein clusters of the different genomes was exported and analyzed via Non Multivariate Analysis (NMDS) based on Euclidean distance. Venn diagram was generated to display the number of core genes. Briefly, we considered as core genome the pool of genes present in all genomes of RIP and NRIP separately. Coding sequences (CDS) for each of the 9 genes were searched through BLASTn^42^ using as query the CDS of *E*. *amylovora* CFBP1430. When partial or no CDS were detected, alignments were manually curated by retrieval of the homologous flanking sequences from the fasta files. DNA polymorphism for each selected gene has been analyzed using the sliding window method with the software DnaSP v6^43^ and expressed as Nucleotide diversity (Pi)^44,45^. The parameters for the analysis have been established in function of the gene length. The window length and the step size values have been settled as 10% and 2.5% respectively (rounded to integer). The Pi value has been calculated for RIP and also for NRIP where the gene was present in a comparable number of species. These outputs have been plotted using Excel. All other graphics, where not explicitly stated, were generated with R^46^.

## Conclusion

The results of this study highlight the presence of a defined sub-group of Rosaceae infecting species taxonomically and genetically related, with a high number of conserved core genes. The importance of the siderophores uptake genes has been extended to the complete transport system of ferrioxamine, with the identification of two genes present exclusively in the strains infecting the Rosaceae. These genes, namely *fhuD* and *sidE*, code for two proteins that require further studies and are possible new targets for development of novel control measures against RIP. Our results raise the interest towards *E*. *tasmaniensis* for a better understanding of the transition between non-pathogenicity and pathogenicity. Moreover, our data confirm the classification of *Erwinia* spp. Ejp617 as *E*. *pyrifoliae*.
